# Characterization of the genomic alterations in poorly differentiated thyroid cancer

**DOI:** 10.1038/s41598-023-46466-5

**Published:** 2023-11-06

**Authors:** Yeeun Lee, SeongRyeol Moon, Jae Yeon Seok, Joon-Hyop Lee, Seungyoon Nam, Yoo Seung Chung

**Affiliations:** 1 Department of Genome Medicine and Science, AI Convergence Center for Medical Science, Gachon Institute of Genome Medicine and Science, Gachon University Gil Medical Center, Gachon University College of Medicine, Dokjeom-ro 3Beon-gil, 38-13, Namdong-gu, Incheon, 21565 Republic of Korea; 2 A.I. Structural Design Team, Division of Biodrug Analysis, New Drug Development Center, OSONG Medical Innovation Foundation, Cheongju-si, 28160 Chungcheongbuk-do Korea; 3 Department of Pathology, Yongin Severance Hospital, Yonsei University College of Medicine, Yongin Severance Hospital 363, Dongbaekjukjeon-daero, Giheung-gu, Yongin-si, Gyeonggi-do 16995 Korea; 4 Department of Surgery, Gachon University Gil Medical Center, Gachon University College of Medicine, Dokjeom-ro 3Beon-gil, 38-13, Namdong-gu, Incheon, 21565 Republic of Korea; 5 Department of Health Sciences and Technology, Gachon Advanced Institute for Health Sciences and Technology (GAIHST), Gachon University, Incheon, 21999 Korea

**Keywords:** Cancer, Genomics, DNA sequencing, RNA sequencing

## Abstract

Poorly differentiated thyroid carcinoma (PDTC) is a subtype of thyroid cancer that has a high rate of metastasis or recurrence and a relatively poor prognosis. However, there are few studies that have been conducted on PDTC at the whole protein-coding gene scale. Here, we performed genomic profiling of 15 patients with PDTC originated from follicular thyroid carcinoma using whole exome sequencing and also performed gene functional enrichment analysis of differentially expressed genes (DEGs) for three patients. Further, we investigated genetic variants associated with PDTC progression and the characteristics of clinical pathology. We revealed somatic genomic alterations in the *RAF1, MAP2K2,* and *AKT2* genes that were not reported in previous studies. We confirmed frequent occurrences in the *RAS* gene in patients with PDTC; the genetic alterations were associated with the RAS-RAF-MEK-ERK/JNK, PI3K-AKT-mTOR signaling pathways, and the cell cycle. DEG analysis showed that immune response was lower in cancer tissues than in normal tissues. Through the association analysis of somatic mutations and the characteristics of clinical pathology from patients with PDTC, the somatic mutations of *ABCA12*, *CLIP1*, and *ATP13A3* were significantly associated with a vascular invasion phenotype. By providing molecular genetic insight on PDTC, this study may contribute to the discovery of novel therapeutic target candidates.

## Introduction

Thyroid cancer is the most common type of endocrine tumor, and its incidence is continuously increasing, and it is the most rapidly increasing cancer globally^1^. More than 90% of thyroid cancer is differentiated thyroid cancer (DTC), which originates from the thyroid follicular epithelial cell; approximately 5% is medullary thyroid cancer (MTC), which originates from the nearby parafollicular C cells^2^. Compared to DTC such as papillary thyroid carcinoma (PTC) and follicular thyroid carcinoma (FTC), which differentiate well by pathological standards and have good prognosis, poorly differentiated thyroid carcinoma (PDTC) and anaplastic thyroid carcinoma (ATC) have a relatively poor prognosis and a higher risk of metastasis or recurrence^1^.

PDTC originates from follicular cells, and its morphology and clinical behavior resemble both DTC and ATC^3^. The term PDTC was previously used to collectively represent various forms of cancer, including solid, trabecular, insular, poorly differentiated, intermediate-type, and primordial cell cancer^4^. However, it has been classified as a new type of cancer by the World Health Organization^4^. Although PDTC is rare, it is very aggressive, and because of its relatively poor prognosis, it is clinically important^3^. Since the diagnosis of PDTC is difficult with the fine needle aspiration biopsy (FNAB) performed prior to surgery, preoperative diagnosis is often delayed^5,6^. It has many similarities to FTC, histologically, and surgery may often be performed twice for treatment^7^. Moreover, because of the characteristics that appear throughout the stepwise dedifferentiation of PDTC^4,8,9^, it shares genomic features with other thyroid cancer subtypes. Therefore, it is difficult to study the genomic characteristics inherent to PDTC^3^.

As the incidence of thyroid cancer increases, there have been active studies on the genomic profiling of patients with thyroid cancer to develop more effective treatment methods^1,10^. With the introduction of next-generation sequencing analysis, the understanding of the genomic landscape of PTC, FTC, and general thyroid cancer has improved^1,10^. In PTC, which is the most common subtype, the *BRAF* alteration occurs at approximately 70% of the time^10^. In FTC, it is known that *RAS (N/H/KRAS)* gene alteration is the most common. However, due to its rare occurrence, sample collection and studies on the analysis of genetic information for PDTC are limited^3^. Despite this, a few studies have attempted to check genomic alterations of PDTC through targeted gene panel sequencing^9,11–13^. There have been a few studies that have examined the genomic landscape of PDTC by whole exome sequencing (WES), but the analyses were performed in few patients^9,14,15^.

In this study, we aimed to identify somatic genomic alterations specific to PDTC and analyzed the WES data on tumor tissue from patients with PDTC against matched normal thyroid tissue to investigate genetic events that impact the known oncogenic signaling pathways. We also identified differentially expressed genes (DEGs) from tumor tissues and the normal tissues nearby through RNA sequencing (RNA-Seq) in three patients; for biological function analysis of DEGs, we performed gene ontology (GO) enrichment analysis and gene set enrichment analysis (GSEA). Lastly, we discovered somatic mutations relevant to tumor progression and clinicopathophysiologic characteristics.

## Results

### WES reveals the genomic landscape of 15 Korean patients with PDTC

**Figure 1:**
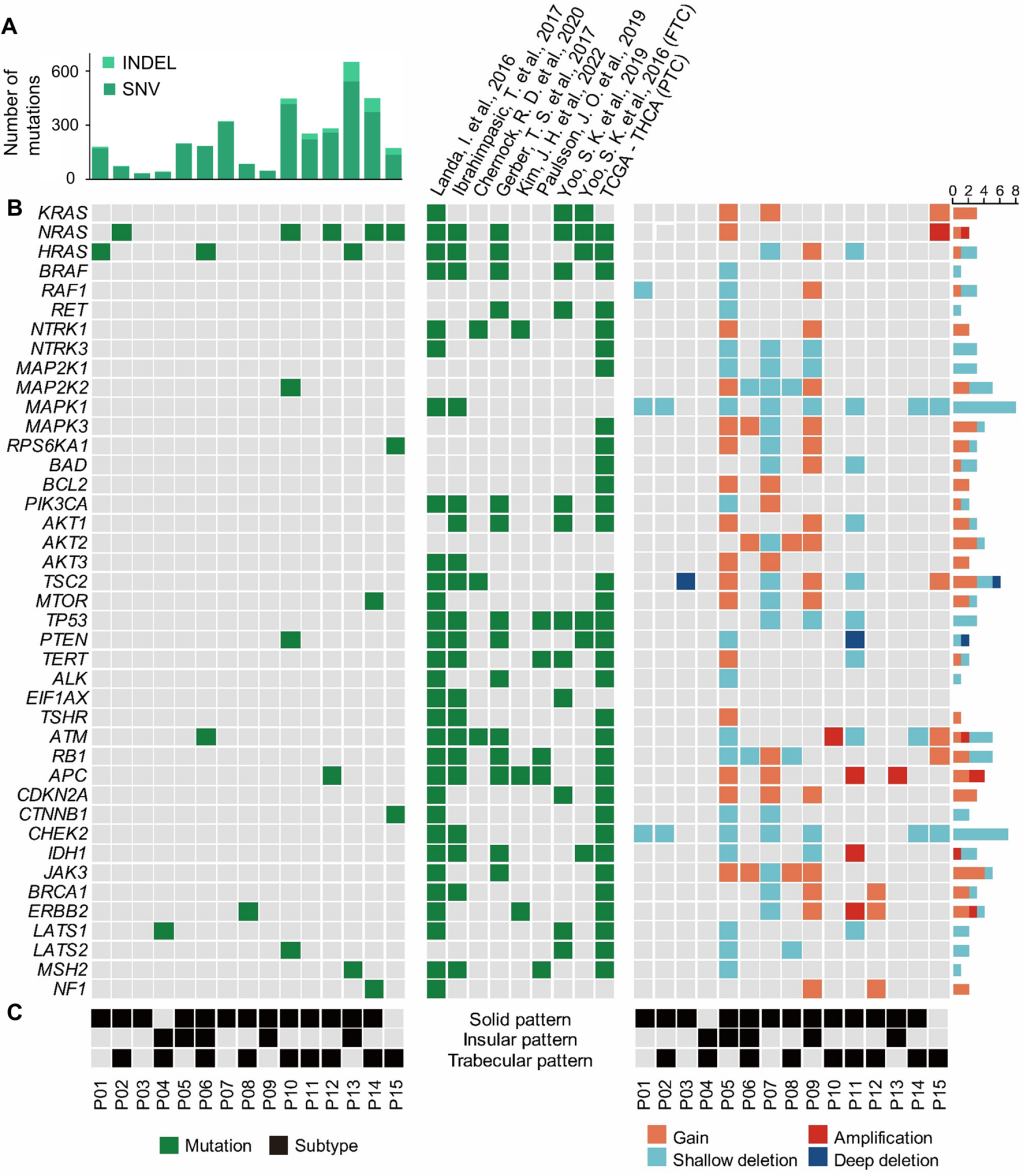
WES reveals the genomic landscape of 15 patients with PDTC and compares them with somatic mutation profiles from published studies. (**A**) Number of somatic mutations in our patients with PDTC. The numbers of SNVs and INDELs for 15 patients with PDTC were depicted. (**B**) Somatic mutation profiles and CNAs of our 15 patients with PDTC were in the left and right panels, respectively. Somatic mutation profiles of previously published studies in PDTC, FTC and PTC were in the middle panel. Genes on the Y-axis belongs to a curated gene set that is reportedly involved in major cancer pathways in thyroid cancer. Columns in the left and right panels refer to individual patients and those in the middle panel individual publications. (**C**) The clinical information on the three subtypes for our 15 patients with PDTC is indicated.

We performed WES on tumor tissues from 15 patients with PDTC originated from FTC and the matched, normal thyroid tissues. We identified 3,446 somatic coding gene mutations (single nucleotide variant (SNV): 3,100; insertion and deletion (INDEL): 346) and copy number alterations (CNAs) in 15 patients with PDTC through WES. The range of mutations per patient was 45-13 (SNV: 32-543, INDEL: 2-108), and the median was 260 (SNV: 184, INDEL: 6; Fig. 1A, Supplementary Tables S1 and S2). For the *RAS* (*H/K/NRAS*) genes, mutations were found in eight patients (53.3%) (Fig. 1B), and this is more frequent than previously reported (28%)^12^. The *BRAF* mutation, whose frequency was reported as 33% in a previous study^12^, was not found in patients in this study (Fig. 1B). The *RAS (H/K/NRAS)’*s copy number gain or amplification was found in four patients (Fig. 1B; Supplementary Figure S1). The WES in our patients also revealed somatic genomic alterations in the *RAF1, MAP2K2* and *AKT2* genes, which were not reported in previous targeted gene panel sequencing in PDTC^9,11–17^ (Fig. 1B). Also, we have examined the predominant patterns in 15 PDTC patients. Among our patients, a solid pattern was observed in 13 patients, an insular pattern in 5 patients, and a trabecular pattern in 9 patients (Fig. 1C).

**Figure 2:**
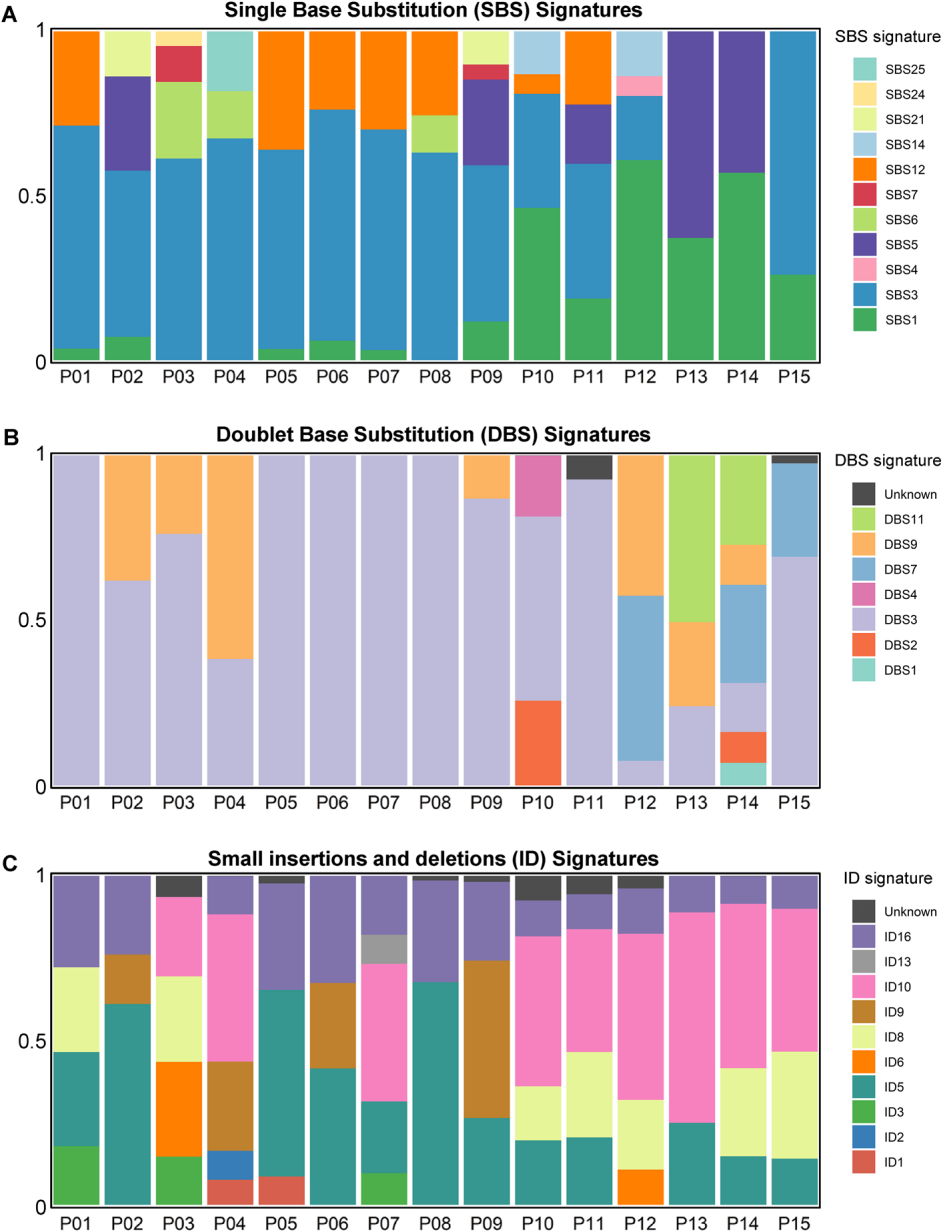
Relative contribution of somatic mutation signatures from 15 patients with PDTC. (**A**) Relative contribution of SBS signatures. SBS1, SBS3, SBS5, and SBS12 were mainly observed in the patients. (**B**) Relative contribution of DBS signatures. DBS3 was contributed to PDTC. **(C)** Relative contribution of ID signatures. ID5, ID10, and ID16 were involved in PDTC.

Mutalisk^18^ was used to confirm the somatic mutation signatures in 15 patients with PDTC at Gil Medical Center (GMC). There was a correlation between age at diagnosis of carcinoma in 12 patients, and the single base substitution (SBS) 1 signature^19^ related to endogenous mutational processes generated from the spontaneous or enzymatic deamination of 5-methylcytosine was identified. Of these, we confirmed that there was a high contribution of > 50% in two patients (Fig. 2A). We confirmed the association with homologous, recombination-based DNA damage repair in 13 patients; SBS3 signatures^19,20^ commonly found in breast, pancreatic cancer, and ovarian cancers were found. The contribution was 50–70% in nine of the 13 patients (Fig. 2A). We noted that SBS5 signature^21^ related to aging, smoking, and nucleotide excision repair (NER) deficiency contributed 15–60% in five patients (Fig. 2A). The SBS12 signature^21^, which contributed a small portion (5–30%) in seven patients, had an unknown cause, and has a low contribution (< 20%) in liver cancer. Of the doublet base substitution (DBS) signatures, DBS3 signature, which is known to be related to the polymerase epsilon exonuclease domain, was found to contribute to all 15 patients (Fig. 2B). Of these, it had a high contribution rate (50–100%) in 11 patients. In addition, the DBS9 signature^22^, which had an unknown cause, contributed some portion (10–55%) in seven patients; DBS11^22^, also with unknown cause, contributed > 50% in 13 patients (Fig. 2B). Most of the insertion and deletion (ID) signatures had unknown causes, and the ID5, ID10, and ID16 signatures^22^, which had major contributions in this cohort of patients, also had no known cause. The ID5 signature that contributed to 12 patients is known to be related to the age at diagnosis. The ID16 signature, which contributed to 14 patients, was identified to contribute to ovarian cancer lacking SBS signatures related to DNA mismatch repair deficiency^22^ (Fig. 2C).

### Comparison between previously known thyroid cancer pathways and the somatic alterations of the patients with PDTC revealed oncogenic signaling pathways

**Figure 3:**
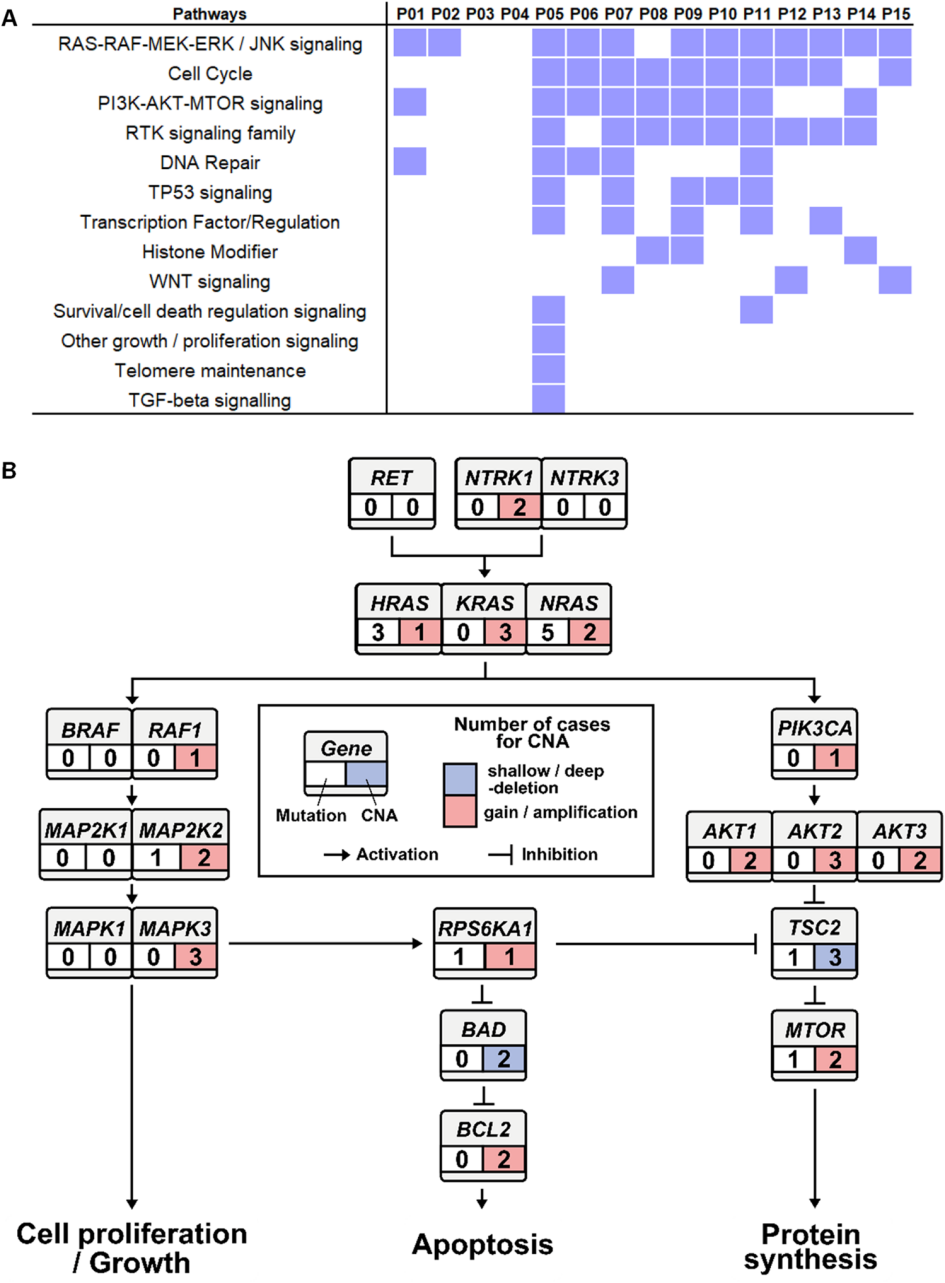
Altered canonical cancer-associated pathways of somatic genomic alterations in patients with PDTC. Somatic genomic alterations (i.e., mutations and CNAs) from the WES data of 15 patients with PDTC were mapped against the major cancer pathways, and significant genomic events were observed. (**A**) Somatic genomic alterations from 13 canonical cancer-associated pathway genes^23^ were confirmed from WES data of 15 patients with PDTC at GMC; genomic events frequently occur at the RAS-RAF-MEK-ERK/JNK signaling pathway. (**B**) Somatic genomic alterations from 15 patients with PDTC at GMC were mapped against the commonly known thyroid cancer oncogenic pathways (BRAF and MAPK pathways) and the alteration of the *RAS* gene was found to be located upstream of the RAS-RAF-MEK-ERK/JNK signaling pathway. The numeral indicates the number of cases having the somatic alterations for the gene. For example, copy number gain/amplification for *BRAF* was observed in one patient, and mutations for the gene were not observed in the patients.

To confirm the oncogenic pathways relating to the patients with PDTC at GMC, we annotated the somatic genomic alterations (i.e., mutations and CNAs) of the patients in 13 canonical cancer-associated pathways^23^ (Fig. 3A). A mutated pathway that was found in most of the patients was ‘RAS-RAF-MEK-ERK/JNK signaling’, and somatic genomic alterations in the pathway were confirmed in 12 of the 15 patients. In addition, somatic genomic alterations were found in more than 60% of patients for ‘cell cycle’, ‘PI3K-AKT-MTOR signaling’, and ‘RTK signaling family’ pathways. Interestingly, two patients (P03 and P04) did not have any somatic genomic alterations of any of the 13 canonical cancer-associated pathways. The ‘RAS-RAF-MEK-ERK/JNK signaling’ and ‘PI3K-AKT-MTOR signaling’ pathways are typical oncogenic signaling pathways in thyroid cancer^24^. In order to compare known thyroid cancer oncogenic pathways with somatic genomic events of the patients with PDTC, we mapped mutations (SNVs and INDELs) and CNAs from 15 patients with PDTC to the oncogenic pathways known for thyroid cancer (Fig. 3B). Thyroid cancer is known to affect the MAPK and PIK3CA/AKT pathways, which contribute to cell proliferation and growth through the RAS signaling pathway^25^. Upon analyzing the pathways, 13 of the 15 patients were found to be affected by the known thyroid cancer oncogenic pathways, and 10 of these patients had somatic mutations, copy number gain or copy number amplification of the *RAS* (*H/K/NRAS*) gene, which are upstream of the MAPK and PIK3CA/AKT pathways (Fig. 3B).

### Functional analysis of DEGs for PDTC tumor tissue and the nearby normal tissues show changes in functions related to cell adhesion and immune response in PDTC

**Figure 4:**
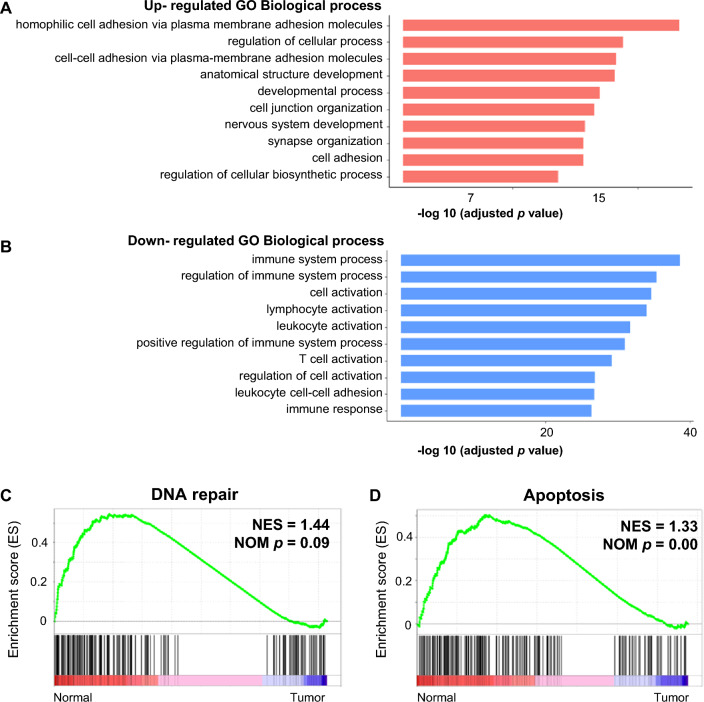
Differentially expressed Genes in PDTC are associated with cell adhesion and immune response. Results from the GO enrichment analysis and GSEA performed using RNA-seq data from three patients with PDTC. (**A**) GOBP enrichment analysis of DEG that are upregulated in PDTC tumor tissue compared to the matched, normal thyroid tissue. They are enriched in terms related to cell adhesion. (**B**) GOBP enrichment analysis of DEG downregulated in PDTC tumor tissue compared to the matched, normal thyroid tissue. They are commonly enriched in terms related to the immune response. The graphic shows the top ten enriched (*p* < 0.05) GOBP terms, starting with the lowest *P*-value. (**C–D**) GSEA results for the hallmark gene sets using RNA-seq data from three patients with PDTC. The graphic shows a significant negative association for DNA repair (**C**) and apoptosis (**D**) for tumor tissues compared to the matched, normal thyroid tissue.

To analyze the function of DEGs in patients with PDTC, we performed GO enrichment analysis and GSEA. Compared to the matched, normal thyroid tissue, 4,668 genes (2,224 genes upregulated, 2,444 genes downregulated) from tumor tissues in three of the patients with PDTC at GMC were significantly differentially expressed. We used g: Profiler tools^26^ to separate the GO of the DEGs (Fig. 4 and Supplementary Figure S2). According to the biological process (BP) term analysis, DEGs upregulated in the tumor tissues (compared to the matched, normal thyroid tissues) were enriched in terms that are related to cell adhesion, such as ‘homophilic cell adhesion via plasma membrane adhesion molecules’, ‘cell–cell adhesion via plasma-membrane adhesion molecules’ or in the terms related to normal cell activity and development, such as ‘regulation of cellular process’ and ‘developmental process’ (Fig. 4A). Compared to the matched, normal thyroid tissue, DEGs downregulated in the tumor tissues were mainly enriched in the terms related to immune responses, such as ‘immune system process’, ‘regulation of immune system process’, and ‘T cell activation’ (Fig. 4B). We performed GSEA to evaluate the difference in the transcription profiles between tumor tissues in patients with PDTC and the matched, normal thyroid tissues. Compared to the matched, normal thyroid tissue, gene sets upregulated in tumor tissues were not found; 15 gene sets, including ‘DNA repair’ and ‘apoptosis’, were downregulated in tumor tissues compared to the matched, normal thyroid tissue (Fig. 4C). Furthermore, we evaluated potential immune checkpoint blockade (ICB) responses in the three patients using the Tumor Immune Dysfunction and Exclusion (TIDE) algorithm^27,28^ (Supplementary Table S3). All three patients were predicted as non-responders.

### Neoantigens were predicted from somatic mutations in PDTC

**Figure 5:**
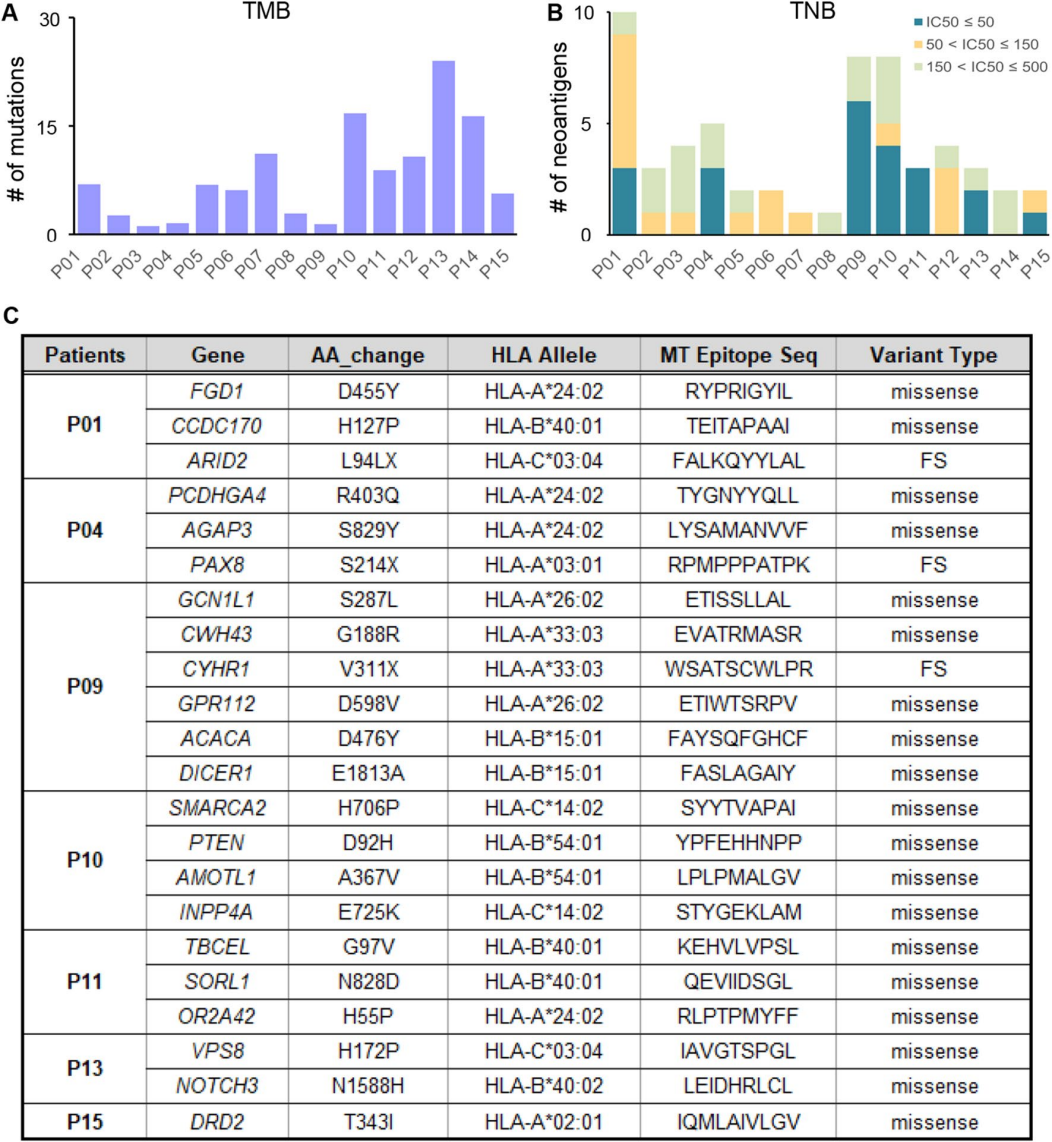
Characteristics of neoantigens predicted from somatic mutations in PDTC. Results of neoantigen prediction analysis using WES data from 15 patients with PDTC at GMC. (**A**) TMB in this patient population is represented. (**B**) The number of neoantigens that have MHC binding affinity < 500 nM in each patient is represented. The shaded area represents MHC binding affinity. **C** List of neoantigens that have a strong affinity (IC_50_ < 50 nM) with MHC predicted from patients with PDTC.

Tumor neoantigen burden (TNB) can affect the response to immune checkpoint inhibitor (ICI) treatment in cancer patients^29^. Tumor mutation burden (TMB) has a correlation with TNB^30^, and it is related to the prognosis of PTC^31^. The average TMB (somatic nonsynonymous mutation counts per Mb) in our patients with PDTC was 6.05 (median 4.89, range: 0.95–17.13; Fig. 5A), and it was higher than TCGA PTC (average 0.41)^1^. In order to explain the conditions for neoantigens in PDTC, we extracted neoantigens with binding affinities of < 500 nM using pVACseq^32^ from the WES data of 15 patients with PDTC at GMC. Of the 2,825 mutations (SNV and INDEL) found in the 15 patients, 58 (1.7%) had neoantigens (IC50 < 500 nM), and there were 54 neoantigens from SNV and four neoantigens from INDEL mutations. With respect to the binding affinity with MHC, there were 22 neoantigens extracted with a high affinity (< 50 nM), 17 neoantigens with a medium affinity (< 150 nM), and 19 neoantigens with a low affinity (< 500 nM; Fig. 5B). Of the neoantigens with a strong affinity, four originated from tumor related genes (*ARID2, DICER1, PAX8*, and *PTEN*; Fig. 5C).

### Somatic mutations of PDTC are potentially related to the clinicopathological characteristics

**Figure 6:**
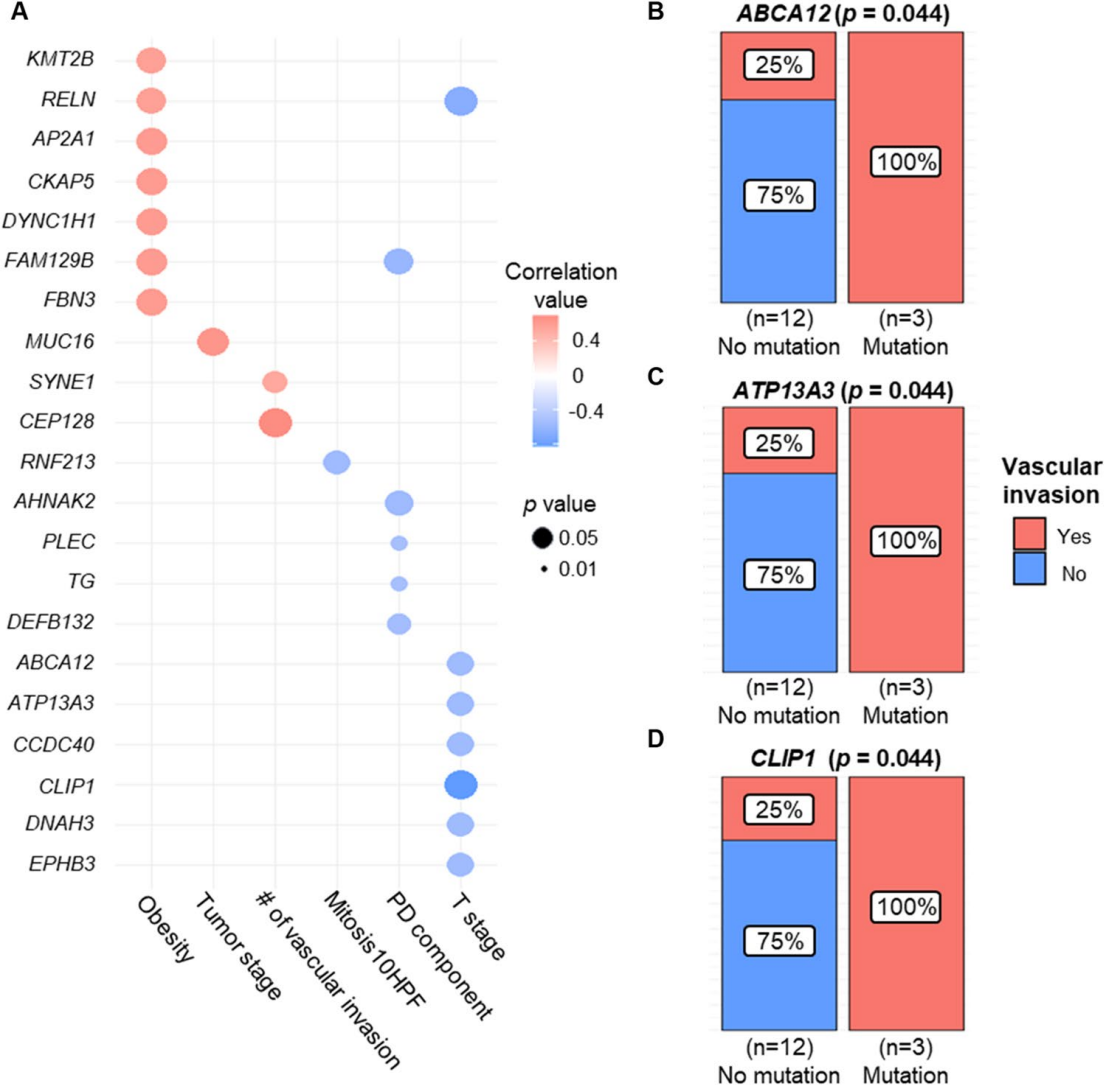
Somatic mutation genes potentially related to PDTC clinicopathological characteristics. We performed association analysis between clinicopathological characteristics of 15 patients with PDTC and the somatic mutation genes. **A** Clinicopathological characteristics and somatic mutation genes with a statistical significance (*p* < 0.05) using one-way ANOVA results. The Y-axis indicates the somatic mutation genes and the x-axis the clinicopathological characteristics. # of vascular invasion, number of foci of vascular invasions; Mitosis10HPF, Mitotic count per 10 high power fields; PD component, the proportion of poorly differentiated components in total tumor tissue. (**B–D**) Clinicopathological characteristics and somatic mutation genes that have a statistical significance (*p* < 0.05) using Fisher’s exact test. Vascular invasion and *ABCA12* (**B**), *ATP13A3* **(C)**, *CLIP1* (**D**) are significantly correlated.

One-way ANOVA and Fisher’s exact test were performed to analyze the relationship between clinicopathological somatic mutation genes from 15 patients with PDTC at GMC. Analysis was performed for genes (n = 100) that had somatic mutations in at least three patients. Mutation status and clinicopathological characteristics were analyzed using one-way ANOVA, and six clinicopathological phenotypes were significantly related to 21 genes (Fig. 6A). Fisher’s exact test showed that four clinicopathological phenotypes were related to nine genes (*p* < 0.05; Figs. 6B, 6C, and 6D; Supplementary Figure S3). *ABCA12, CLIP1,* and *NRAS* were related to trabecular pattern (Supplementary Figures S3A, B, and C); *ABCA4* and *SLIT3* were related to insular pattern (Supplementary Fig. S3D and E); *ABCA12*, *ATP13A3*, and *CLIP1* genes were related to the status of vascular invasion (Figs. 6B–D); *INTS1* and *RYR1* genes were related to the status of capsular invasion (Supplementary Figure S3F and G); and the *SORL1* gene was related to the status of hypertension (Supplementary Figure S1H).

## Discussion

Our study aimed to shed light on the genetic landscape of PDTC, an area with limited existing knowledge. To achieve this, we conducted WES and RNA-Seq to uncover the molecular genetic characteristics of PDTC and to explore their associations with the disease. To comprehensively analyze these aspects, we employed various bioinformatics tools and methodologies, including mutation profile analysis, oncogenic pathway analysis, assessment of tumor mutation burden, prediction of neoantigens, DEG analysis, gene ontology enrichment analysis, and investigations into the associations between clinical parameters and somatic mutation profiles. It is worth noting that our study stands out in terms of WES analysis by utilizing data from 15 patients of PDTC originated from FTC, representing a relatively large sample size compared to previous PDTC WES studies^9,14,15^. This substantial dataset allowed us to draw robust conclusions and identify potential clinical implications.

In this study, the *RAS* gene mutation was frequently seen in our patients, and the *BRAF* mutation^12^, which is typically known in thyroid cancer, was not seen in our patients. All of the PDTC in our patient population originated from FTC, and FTC has a characteristic of low *BRAF* gene mutation frequencies^33^. Genomic events were found in the thyroid cancer, MAPK via RAS signaling, and PIK3CA/AKT signaling pathways in 13 patients^25^.

In Fig. 1B, the *RAS (H/N/KRAS)* genes are oncogenes, which are often mutated. The genes are related to cell proliferation and survival^33^. *RAS* genes are located at the upstream of the MAPK and PIK3CA/AKT signaling pathways. The MAPK pathway mutation is found in most cancers, and the PIK3CA/AKT pathway is known to be upregulated in thyroid cancer^34^. In Fig. 1B, *MAP2K2* is a gene that encodes mitogen-activated protein kinase kinase 2 (MEK2) and regulates cell growth and proliferation by phosphorylating ERK1/2 in the MAPK pathway, thereby activating it^35^. We found a somatic mutation or copy number gain in *MAP2K2* in three patients (P05, P09, and P10 in Fig. 1B). It is noted that the somatic mutation in *MAP2K2* was not previously reported in targeted gene panel sequencing of PDTC^9,11–13^.The *MAP2K2* mutation is related to a high resistance in MEK inhibitors in melanomas with a *BRAF* mutation^35^, and *MAP2K2* upregulation is known to induce the progress of clear cell renal carcinoma^36^. However, its oncogenic role in thyroid cancer is unknown.

Cancer immune escape is the most important barrier in improving cancer treatment strategies^37^. Based on the results of GO enrichment analysis using RNA-Seq data from three patients (P02, P03, and P04) with PDTC, genes that were downregulated in PDTC tumor tissues compared to normal thyroid tissues were enriched in the terms related to the immune responses of GO biological processes (GOBP in Fig. 4B). All three patients demonstrated relatively low TMB and high TIDE scores, implying a higher likelihood of tumor immune evasion. This suggests a lower probability of benefiting from treatment with immune checkpoint inhibitors^27,28^. Immune response-related genes that were downregulated in the tumor tissues of patients with PDTC compared to normal thyroid tissues included *TAP1, AIM2,* and *MICB. TAP1* downregulation is associated with immune evasion and unfavorable prognosis in rectal cancer^38^, and *AIM2* is a known tumor suppressor gene in melanoma^39^. *MICB* is a gene that codes the major histocompatibility complex class I related B (MIC-B), and it is related to the innate immune system^40^. *MIC-B* downregulation is the cancer cell immune evasion strategy to avoid NK-mediated cytotoxic killing^41^, and *MIC-B* downregulation is associated with poor prognosis of colorectal cancer^42^.

TMB is known as a biomarker for immune checkpoint blockade (ICB) response^43^. Well-differentiated thyroid cancers, such as PTC and FTC, exhibit relatively lower TMB compared to other cancer types^44^. Progressive thyroid cancer subtypes like PDTC have higher TMB, with an even higher TMB observed in ATC^44^. Previous studies have proposed an optimal TMB cut-off (> 13 mut/Mb) for ICB response. Among our 15 patients, three (P10, P13, and P14) exceeded this cut-off, each having TMB values of 16.8, 24.0, and 16.4 mut/Mb, respectively, indicating likely hyper-mutated cases. According to previous ATC research, such a 'hypermutator phenotype' may arise in the presence of mutations in DNA mismatch repair (MMR) genes^45^, and one (P13) of our three patients indeed had a mutation in a DNA MMR gene, *MSH2*.

In Fig. 5, we detected neoantigens that originated from four cancer related genes (*ARID2, DICER1, PAX8,* and *PTEN*) using neoantigen prediction from WES data and 22 neoantigens had strong affinity. *ARID2* is a tumor suppressor gene, and its mutants occur in various cancer types, including stomach cancer, melanoma, and colorectal cancer^46^. Tumors with *ARID2* mutants can potentially be more sensitive to immune-checkpoint inhibition^47^. *DICER1* is an important tumor suppressor gene in thyroid cancer^48^, and its mutants have been reported in thyroid cancer^1,9^. *PAX8* is an important gene in thyroid cell development^49^, and PAX8/PPARγ fusion protein from the translocation is a driver mutation that is often seen in FTC^33^. *PTEN* is a tumor suppressor gene that is commonly mutated in human cancers^50^. Loss of *PTEN* function can contribute to the development of cancer by activating PI3K/AKT/mTOR signaling^50^.

Recurrence and metastasis occur more commonly in PDTC relative to FTC or PTC^8^. It has been reported that patients with PDTC with vascular invasion have a higher chance of recurrence or metastasis in comparison to patients with PDTC without vascular invasion^51^, and we confirmed that six patients with PDTC had vascular invasion (Fig. 6B). Given the significant correlation observed in the association analysis (Fig. 6B) between *ABCA12*, *ATP13A3*, and *CLIP1* somatic mutations and vascular invasion, these somatic mutations may hold promise as potential biomarkers for predicting the recurrence or metastasis of PDTC following surgery. Notably, *ABCA12* knockdown resulted in decreased cell motility, proliferation, and invasion in pancreatic cancer cells, such as SW199^52^. Among the observed *ABCA12* mutations in three patients (P01, P05, and P07), the presence of these mutations suggests a potential gain-of-function effect on cell motility.

*ATP13A3* was recently suggested to be a major mammalian polyamine transporter, and it was found to be expressed at a high level in pancreatic cancer patients, thereby reducing survival rates^53^. CLIP1 encodes a protein that controls the dynamics of microtubule cytoskeleton in cells^54^ and its mutations are known carcinogenic factors in non-small-cell lung cancer^54^.

One limitation of our study is the small sample size for RNA-Seq analysis. We conducted gene expression profiling in only three patients (P02, P03, and P04), who exhibited relatively lower TMB when compared to the remaining 12 patients (Figs. 1A and 3A). Consequently, the gene expression profiling findings in our study might not accurately reflect the characteristics of Korean patients with PDTC. Therefore, it is advisable to exercise caution when interpreting these results. Nevertheless, the DEG ontology analysis performed in the study may expand the understanding of PDTC. Validation of the findings through larger cohort studies and additional studies for the profiling of tumor from single cell RNA-Seq will be needed in the future.

In our patients, genetic alterations within known oncogenic pathways were observed to occur more frequently in copy number alterations (CNAs) than in point mutations. This suggests the potential involvement of CNAs as driver events in tumorigenesis^55^. However, we were unable to simultaneously evaluate both copy number changes and alterations in gene expression levels. Previous studies have indicated a higher likelihood of genes with DNA copy number amplification and elevated gene expression levels being associated with oncogenes^55^. Validation of our findings will be necessary through larger-scale cohort studies and further research involving multi-omics tumor profiling, including single-cell RNA-Seq, in the future.

## Conclusion

In conclusion, we identified that somatic mutation genes in patients with PDTC are associated with the clinicopathological characteristics of the tumor, and we confirmed the possibility of immune escape through transcriptome analysis. These findings provide insights into novel treatment strategies for PDTC.

## Materials and methods

### Study subjects and patients

This study included 15 patients with PDTC originated from FTC at Gachon University Gil Medical Center (GMC), South Korea. Primary tumor and matched, adjacent normal thyroid tissue were collected from the 15 patients for mutation profiling and mRNA expression profiling. Clinicopathological characteristics of the patients are summarized in Table 1. Extrathyroidal extension was found in one patient, and capsule formation was found in 14 patients. Ten patients had capsular invasion, and vascular invasion was confirmed in six patients. None of the 15 patients had lymph node metastasis.

**Table 1:** Clinicopathological characteristics.

Patient characteristics	N
Age	
≥ 60	5
< 60	10
Sex	
Female	10
Male	5
Mean tumor size (range), cm	4.8 (1.3–9.5)
Extrathyroidal extension	1
Capsule formation	14
Capsular invasion present	10
Vascular invasion present	6
Lymph node metastasis	0
Solid pattern	13
Insular pattern	5
Trabecular pattern	9
TNM stage	
I/II	13
III/IV	2

The study was approved by the GMC Ethics Committee (GBIRB2020-098) and conducted in accordance with the Declaration of Helsinki. All participants provided written informed consent.

### DNA extraction and sequencing analysis

For mutation profiling in 15 patients with PDTC, whole-exome sequencing (WES) was performed using tumor tissue and matched, normal thyroid tissue. The SureSelect XT V6 Library Prep Kit (Agilent, Santa Clara, CA, USA) was used as the exome library, and sequencing was performed using the Illumina sequencing platform (Macrogen Inc., Seoul, Korea; Supplementary Table S4).

Sequenced data (FASTQ) was trimmed (q > 30) according to the quality score using Sickle (v1.33) and was aligned for hg19 using BWA-mem (v0.7.15). Duplicate reads were confirmed and edited using Picard MarkDuplicates (v2.5.0). Using the Genome Analysis Toolkit (v3.6)^56^, sorting errors from insertion and deletion (INDEL) were corrected and the quality score was edited. For BAM files, which were realigned and edited, Mutect2^56^ was used along with the matched, normal thyroid tissue to call for the somatic single nucleotide variants (SNV) and INDEL. To remove low quality variants, we selected data with the ‘PASS’ label from the results of Mutect2. Somatic SNV and INDELs were annotated using Annovar (v2016-02-02).

To find somatic mutations, variants with ≥ 1% minor allele frequency (MAF) in more than one of the three germline population variant’s databases (1000 Genomes Phase 3, ESP6500, ExAC) were removed. Finally, to select for the somatic coding mutation, we selected missense, nonsense, and INDEL mutations from the exon and splice sites. Tumor mutation burden was calculated using the somatic nonsynonymous mutation (SNV, indel) per mega base in the coding region (exon and splice region)^1^.

### Copy number alterations (CNAs)

According to the workflow recommended by VarScan (v2.4.4)^57^, somatic CNAs were recalled from WES BAM files. Using the online software package, GenePattern GISTIC 2.0 tools, genomic level CNAs and recurrent events in the cohort were identified.

### RNA sequencing analysis

RNA-Seq was performed using tumor tissue from three GMC patients with PDTC and matched normal thyroid tissue (Supplementary Table S5). An RNA-Seq library was prepared using the SureSelectXT RNA Direct Reagent Kit (Agilent, Santa Clara, CA, USA), and it was sequenced using the Illumina platform (Illumina, San Diego, CA, USA; Supplementary Table S6). The sequences were mapped against the known reference genome using the HISAT2 program^58^, and transcript assembly was done using the StringTie. DEG analysis was performed using DESeq2.

### Collection of publicly available PDTC and PTC data sets for comparison of mutation profiles

We collected mutation profile data from patients with PDTC and PTC available on cBioPortal^59^. Mutation profiles from the 341 major cancer genes of 84 patients with PDTC provided by the Memorial Sloan Kettering Cancer Center (MSKCC) data set^12^ were collected. From The Cancer Genome Atlas (TCGA) data set^1^, in which WES was performed in 315 patients with PTC, mutation profiles were collected. Additionally, we collected mutation profiles from previous studies that performed target sequencing in patients with PDTC^9,11–13^.

### Mutational signature analysis

Using Mutalisk^18^, which is a somatic mutation analysis tool, single base substitution (SBS) signatures were confirmed. The SBS signature was dissembled using the SBS signature (v3.3) provided by the Catalogue of Somatic Mutations in Cancer (COSMIC)^60^. The relative contribution of the SBS signature was calculated using linear regression for all 78 SBS signatures. The variant matrix for doublet base substitution (DBS) signatures and small insertion and deletion (ID) signatures was composed using ‘SigProfilerMatrixGenerator’, and the contributions to DBS Signatures (v3.3) and ID signatures (v3.3) provided by COSMIC were calculated using the ‘deconstructSigs (v1.9.0)’ package in R software.

### Human leukocyte antigen (HLA) typing and neoantigen prediction

Using the Polysolver program^61^, the HLA type was determined from the human leukocyte antigen (HLA) class I gene (HLA-A, HLA-B, HLA-C) of 15 patients with PDTC at 4 × resolution. In order to predict the neoantigens, the pVAC-Seq pipeline^32^ was used for analysis using the variant call format (VCF) file, which has ensemble variant effect predictor (VEP) annotation. Major histocompatibility complex (MHC) binding affinity for the whole peptide was estimated using the binding prediction algorithms for the pVAC-Seq pipeline, NetMHC and NetMHCpan (< 500 nM binding affinity). The pVec-Seq parameters were set as default, and the peptide length for the measurement of MHC and binding affinity was set as 8–10 amino acid (aa). We selected peptides that were 8–10 aa in length with < 500 nM for MHC and binding affinity, as well as those that appeared only in tumor tissues, as neoantigen candidates. We defined neoantigens with a binding affinity of < 50 nM as ‘strong affinity’, those with a binding affinity of < 150 nM as ‘medium affinity’, and those with a binding affinity of < 500 nM as ‘weak affinity’.

### Gene ontology (GO) enrichment analysis and gene set enrichment analysis (GSEA)

For the biological functional annotation of significantly differentially expressed genes from the tumor tissues of three patients with PDTC against the matched, normal thyroid tissue, we used the ‘g:Profiler’ tool^26^ to perform GO enrichment analysis. Using tumor tissue from three patients with PDTC and the matched, normal thyroid tissue gene expression profiles, we performed gene set enrichment analysis (GSEA, v4.2.2)^62^ for functional annotation. For the reference gene set, the hallmark gene set (v7.5.1)^62^ was used, which includes the well-defined biological states or processes provided by the Molecular Signatures Database (MSigDB)^62^; all variables were set as default. We determined a False Discovery Rate (FDR) of < 0.25 as statistically significant^62^.

### Prediction of the immune checkpoint blockade (ICB) response

Predicting the Immune Checkpoint Blockade (ICB) Response the Tumor Immune Dysfunction and Exclusion (TIDE)^28,63^ was developed to predict potential immune checkpoint blockade (ICB) responses. Using RNA-Seq data from three patients, we predicted ICB responses following the procedures outlined on the TIDE website in order to evaluate their response to immunotherapy.

### Clinical correlation analysis between mutation profiles and clinicopathological data

To find somatic mutations that have significant statistical relevance to clinicopathological characteristics, we analyzed mutations profiles from 15 patients with PDTC and the clinicopathological data using Fisher’s exact test and one-way ANOVA. A *P*-value < 0.05 was considered as statistically significant.

### Ethics approval and consent to participate

The study was approved by the GMC Ethics Committee (GBIRB2020-098) and conducted in accordance with the Declaration of Helsinki.

### Supplementary Information

Supplementary Information 1.

Supplementary Information 2.

Supplementary Information 3.

## Data Availability

The WES and RNA-Seq data generated in this study are deposited in the NCBI Sequence Read Archive (SRA) Database (PRJNA883015).
