# Supplementary material for: Characterization of the genomic alterations in poorly differentiated thyroid cancer
Source: Sci Rep. 2023 Nov 6;13:19154. doi: 10.1038/s41598-023-46466-5 (PMC10628257; doi:10.1038/s41598-023-46466-5)
Supplement: Supplementary file 1 — Supplementary Information 1. [file 41598_2023_46466_MOESM1_ESM.pdf]

Supplementary Information

Characterization of the genomic alterations in poorly differentiated thyroid cancer

Yeeun Lee, SeongRyeol Moon, Jae Yeon Seok, Joon-Hyop Lee, Seungyoon Nam, and Yoo Seung Chung

Contents

Supplementary Figures 1 through 3.

Supplementary Tables 1 through 6.

Note: Supplementary Tables 2 and 5 are provided in excel format.

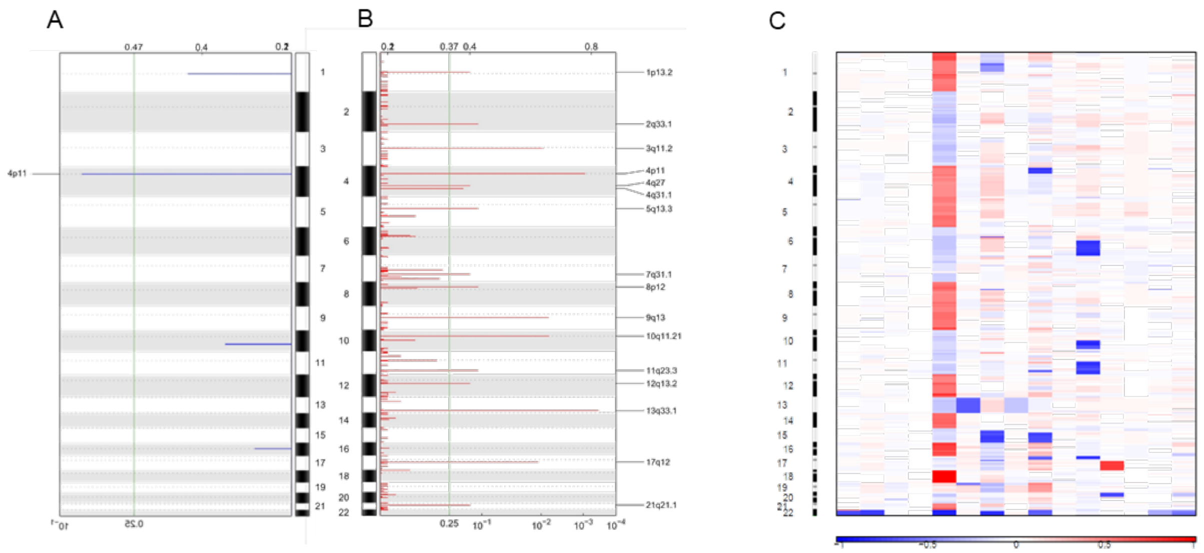

Supplementary Figure 1. CNA analysis of 15 PDTC patients. (A) One deleted focal region was identified in 15 patients. **(B)** 16 amplified focal regions were identified in 15 patients. The GISTIC2.0 q-values are plotted on a log scale, and the green line indicates the significance threshold (q-value 0.25). **(C)** The heat map by GISTIC 2.0 (https://broadinstitute.github.io/gistic2/) shows the amplified (red) and deleted (blue) regions along the chromosome.

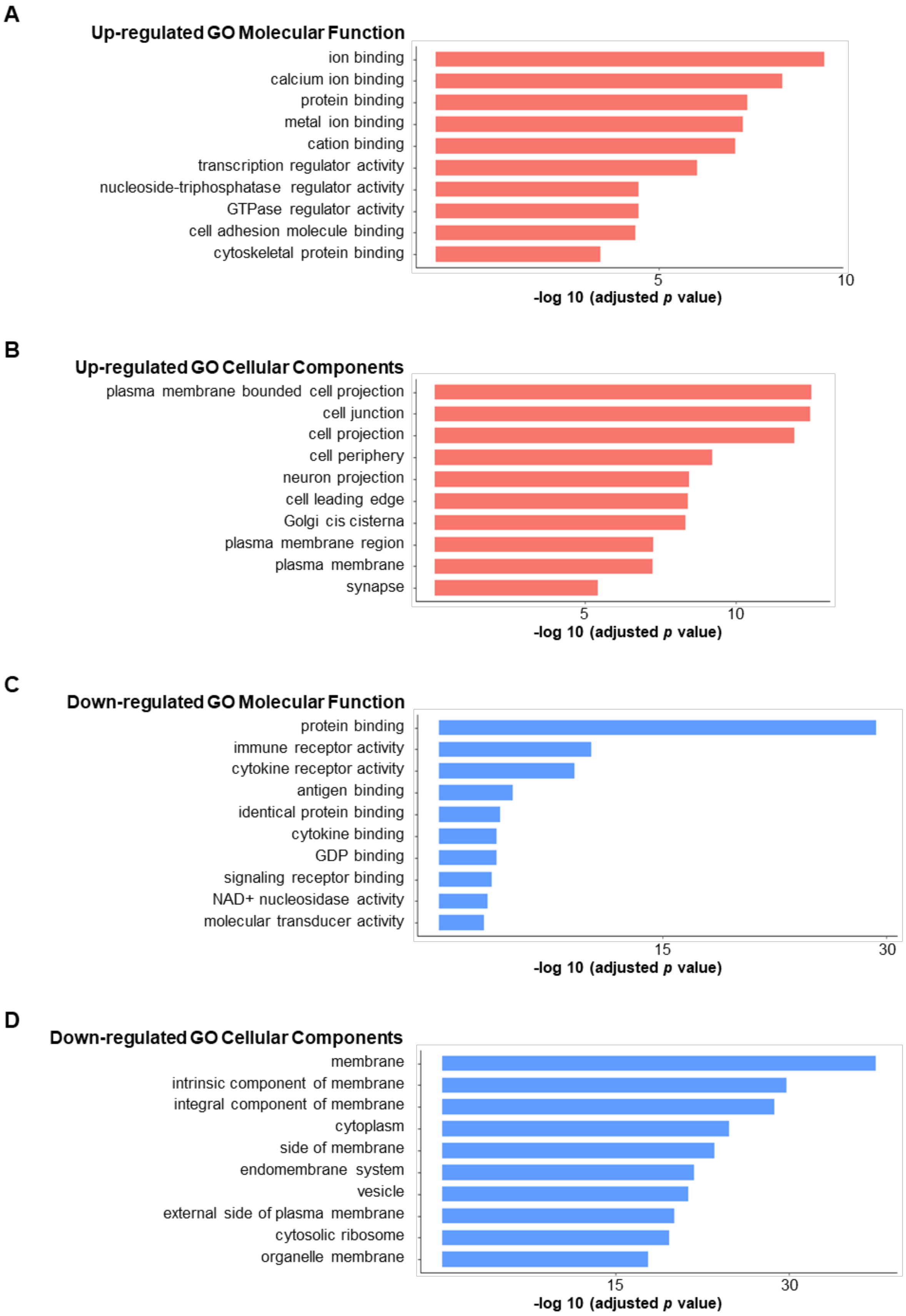

A
Up-regulated GO Molecular Function
ion binding
calcium ion binding
protein binding
metal ion binding
cation binding
transcription regulator activity
nucleoside-triphosphatase regulator activity
GTPase regulator activity
cell adhesion molecule binding
cytoskeletal protein binding
5
10
-log 10 (adjusted p value)
B
Up-regulated GO Cellular Components
plasma membrane bounded cell projection
cell junction
cell projection
cell periphery
neuron projection
cell leading edge
Golgi cis cisterna
plasma membrane region
plasma membrane
synapse
5
10
-log 10 (adjusted p value)
C
Down-regulated GO Molecular Function
protein binding
immune receptor activity
cytokine receptor activity
antigen binding
identical protein binding
cytokine binding
GDP binding
signaling receptor binding
NAD+ nucleosidase activity
molecular transducer activity
15
30
-log 10 (adjusted p value)
D
Down-regulated GO Cellular Components
membrane
intrinsic component of membrane
integral component of membrane
cytoplasm
side of membrane
endomembrane system
vesicle
external side of plasma membrane
cytosolic ribosome
organelle membrane
15
30
-log 10 (adjusted p value)

Supplementary Figure 2. GO enrichment analysis for DEG of 3 PDTC patients. (A) Up-regulated GO molecular function in patients with PDTC. **(B)** Up-regulated GO cellular components in patients with PDTC. **(C)** Down-regulated GO molecular function in patients with PDTC. **(D)** Down-regulated GO cellular components in patients with PDTC.

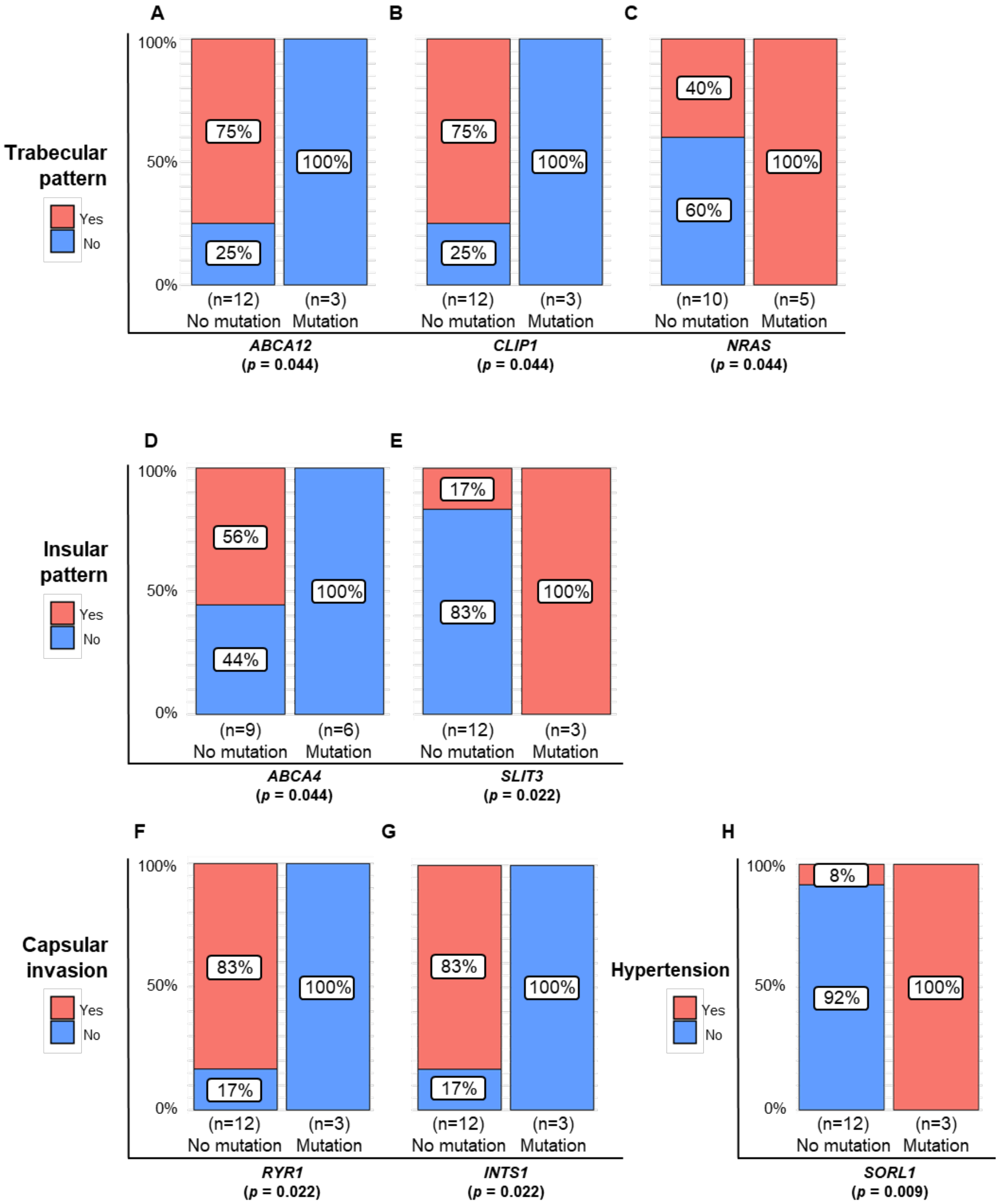

Supplementary Figure 3. Correlation analysis with clinical and genomic variables. Somatic mutation genes correlated with clinicopathological characteristics (Fisher's exact test $p<0.05$). **(A-C)** Trabecular pattern significantly correlated with *ABCA12* (A), *CLIP1* (B), and *NRAS* (C). **(D, E)** Insular pattern significantly correlated with *ABCA4* (D) and *SLIT3* (E). **(F, G)** Capsular invasion significantly correlated with *RYR1* (F), and *INTS1* (G). **(H)** Hypertension significantly correlated with *SORL1*.

Supplementary Table 1. Statistics of somatic genetic alterations for 15 patients with PDTC.

Patients	Non-synonymous SNVs (including missense, stop-gain/loss, and splicing)	Synonymous SNVs	Indels	Total
**P01**	173 (65.53 %)	82 (31.06 %)	9 (3.41 %)	264
**P02**	71 (70.29 %)	25 (24.75 %)	5 (4.95 %)	101
**P03**	32 (71.11 %)	9 (20.00 %)	4 (8.89 %)	45
**P04**	39 (65.00 %)	15 (25.00 %)	6 (10.00 %)	60
**P05**	199 (76.54 %)	59 (22.69 %)	2 (0.77 %)	260
**P06**	184 (78.97 %)	47 (20.17 %)	2 (0.86 %)	233
**P07**	319 (74.88 %)	103 (24.18 %)	4 (0.94 %)	426
**P08**	84 (76.36 %)	24 (21.82 %)	2 (1.82 %)	110
**P09**	47 (87.04 %)	5 (9.26 %)	2 (3.70 %)	54
**P10**	417 (65.46 %)	189 (29.67 %)	31 (4.87 %)	637
**P11**	222 (65.68 %)	84 (24.85 %)	32 (9.47 %)	338
**P12**	260 (63.41 %)	127 (30.98 %)	23 (5.61 %)	410
**P13**	543 (59.47 %)	262 (28.70 %)	108 (11.83 %)	913
**P14**	373 (59.97 %)	171 (27.49 %)	78 (12.54 %)	622
**P15**	137 (63.43 %)	41 (18.98 %)	38 (17.59 %)	216

Supplementary Table 3. TIDE results of the 3 patients with PDTC.

Patient	P02	P03	P04
Responder type	Non-responder	Non-responder	Non-responder
**TIDE (score)**	**0.99**	**0.57**	**0.61**
IFNG	-0.32	-0.35	-0.36
MSI Expr Sig	0.29	0.38	0.4
Merck18	-0.37	-0.43	-0.49
CD274	-0.4	-0.82	-0.93
CD8	-0.17	-0.36	-0.37
Dysfunction	0.15	0.06	0.04
Exclusion	0.99	0.57	0.61
MDSC	0.08	0.09	0.08
CAF	0.04	-0.03	-0.01
TAM M2	0.02	0.03	0.02

Supplementary Table 4. Sequencing coverage and quality statistics of WES.

Sample ID	Total number of sequenced reads	Total number of uniquely mapped non-duplicate reads	Total number of covered bases	Mean depth of target regions (X)	Percentage of targeted bases with coverage ≥10
P01N	69,806,642	63,966,145	42,763,851	60.9	97.3
P01T	214,746,264	195,040,930	120,576,900	171.9	99.3
P02N	71,020,288	65,713,330	43,107,830	61.5	97.0
P02T	203,239,666	184,394,266	125,973,769	179.9	99.3
P03N	74,647,410	67,907,125	41,018,715	58.5	97.1
P03T	196,768,664	176,339,032	108,802,086	155.1	99.1
P04N	77,895,902	70,815,480	49,110,201	70.0	97.9
P04T	236,581,226	209,952,429	140,883,665	201.2	99.6
P05N	73,902,932	66,796,651	45,782,812	65.4	97.5
P05T	181,719,980	165,983,228	111,210,403	158.6	99.2
P06N	91,238,038	82,502,276	53,712,352	76.5	98.3
P06T	239,886,850	215,587,071	146,937,322	209.8	99.6
P07N	68,652,396	63,194,336	39,060,785	55.7	96.6
P07T	236,604,920	212,010,133	144,966,512	207.1	99.6
P08N	80,735,168	73,860,203	47,765,176	68.1	97.6
P08T	213,333,618	190,626,444	124,327,535	177.5	99.3
P09N	82,655,238	74,590,327	48,637,447	69.4	97.7
P09T	178,490,656	162,862,337	103,675,268	147.5	99.2
P10N	74,242,634	63,956,029	50,179,711	73.9	87.0
P10T	252,393,364	208,545,437	164,513,724	241.5	98.5
P11N	67,574,922	60,659,083	47,935,469	70.3	84.7
P11T	241,317,330	204,222,445	154,394,374	222.4	99.1
P12N	63,880,228	56,164,393	43,843,871	63.9	89.8
P12T	229,672,362	192,217,423	148,983,508	216.6	97.8
P13N	69,965,644	60,129,190	47,365,655	69.9	85.3
P13T	256,994,646	172,120,070	137,835,643	205.5	93.7
P14N	73,255,754	60,128,332	44,622,240	63.7	95.0
P14T	251,646,208	199,675,662	155,723,226	227.3	98.4
P15N	67,979,022	61,277,312	46,713,792	67.5	90.9
P15T	221,490,324	187,221,935	143,779,848	208.2	99.0

Supplementary Table 6. Sequencing coverage and quality statistics of RNA-Seq.

Sample ID	Total number of sequenced reads	GC content (%)	The ratio of base ≥ Phred quality score 20	The ratio of base ≥ Phred quality score 30
P02N	124,724,446	51.44	98.96	96.69
P02T	114,131,232	54.09	98.93	96.65
P03N	95,749,532	50.11	98.86	96.47
P03T	98,634,842	54.50	98.86	96.55
P04N	95,792,014	49.58	98.92	96.59
P04T	110,869,504	55.62	98.76	96.40
